# Specification and Performance Indicators of AeroRing—A Multiple-Ring Ethernet Network for Avionics Embedded Systems

**DOI:** 10.3390/s18113871

**Published:** 2018-11-10

**Authors:** Ahmed Amari, Ahlem Mifdaoui

**Affiliations:** ISAE-SUPAERO/Université de Toulouse, 10 avenue Edouard Belin, 31055 Toulouse, France

**Keywords:** Sensor Networks, Ethernet, ring, avionics, maximum delivery time, redundancy recovery time, IEC 61784, IEC 62439

## Abstract

The complexity and costs of the avionics communication architecture are increasing exponentially with the increasing number of embedded computers over the last few decades. To limit the cabling complexity and the deployment costs of such a communication architecture, we specify a new Gigabit multiple-ring Ethernet network, called AeroRing, while meeting the avionics requirements. First, we describe the current Aircraft Data Communication Network (ADCN) to highlight the main characteristics and requirements that have to be fulfilled by our solution. Then, we give an overview of the most relevant solutions to improve ADCN performance and relate them to AeroRing. Afterwards, we detail the specifications and the main Performance Indicators (PIs) of AeroRing. Finally, sensitivity and validation analyses of AeroRing are conducted through a realistic avionics application, regarding the various PIs, in comparison to the backbone network of the ADCN, the Avionics Full DupleX Switched Ethernet (AFDX). The computed AeroRing performance metrics show its ability to guarantee the avionics requirements.

## 1. Introduction

Nowadays, the complexity of embedded systems is growing in several domains, such as civil and military avionics, aerospace, and railways. This complexity, particularly in avionics, is due to the increasing number of embedded functions over the last few decades. Since the Airbus A300 in 1972, the amount of embedded electronic equipment is constantly increasing [1] to offer new functionalities, improving performance, safety, and maintenance. This fact leads to an increasing number of embedded computers, sensors, and actuators; thus, the quantity of wires, weight, and costs.

To cope with these emerging avionics needs, high-speed communication networks have been integrated in new-generation aircraft. For instance, the 100 Mbits/s Avionics Full DupleX Switched Ethernet (AFDX) [2] is used as a backbone network, to interconnect the critical avionics systems in the Airbus A380 and A350. However, low-rate buses such as ARINC 429 [3,4] and Controller Area Network (CAN) [5], are still used for the sensors/actuators and cabin communications.

Although this communication architecture meets the main avionics requirements, it leads at the same time to an inherent heterogeneity of the interconnection means and a large amount of cables and connectors, thus high integration costs. The costs related to cabling during the manufacturing and installation are actually between 14 million dollars for an Airbus A320 to 50 million dollars for an Airbus A787 [6]. Moreover, the cabling complexity is considered to be one of the main reasons behind the production delays of the Airbus A380, where the cost overruns have been estimated at 2 billion dollars [6].

To handle these limitations, our main solution is based on a Full DupleX Gigabit Ethernet multiple-ring network, called AeroRing, meeting the avionics requirements while limiting the cabling complexity and the deployment costs. A preliminary version of AeroRing specifications in the case of a simple mono-ring topology has been described in [7], and a comparative analysis of AeroRing with the most relevant ring-based real-time Ethernet solutions [8,9] has been conducted in [10].

Hence, in this paper, our main contributions are as follows:

- Extended specification of AeroRing covering the multiple-ring topology: To satisfy the avionics requirements in terms of scalability, availability, and real-time performance, while keeping the IEEE802.3 compatibility, reducing the implementation costs and configuration efforts.
- Definition of the main Performance Indicators of AeroRing: The analytical formula of the main performance indicators of AeroRing are detailed, as required in the standard IEC 61784 document [9], and particularly the maximum delivery and recovery times, which are of utmost importance to assess the predictability and availability levels of AeroRing vs. the avionics requirements.
- Validation of AeroRing: We validate AeroRing by investigating the offered timing performance and availability levels through a realistic avionics case study of an Airbus A380. The latter consists in replacing the AFDX network consisting of eight AFDX switches connecting 54 end-systems with an AeroRing multiple-ring network. AeroRing performance metrics are compared to the AFDX and show its ability to guarantee the avionics requirements.

The rest of the paper is organized as follows: we first describe the current Aircraft Data Communication Network (ADCN) to highlight the main characteristics and requirements that have to be fulfilled by an alternative solution in Section 2. Then, we give an overview of the most relevant solutions to improve ADCN performance and relate them to our proposal in Section 3. Afterwards, we detail the specifications and the main Performance Indicators (PIs) of AeroRing in Section 4 and Section 5, respectively. Finally, sensitivity and validation analyses of AeroRing are conducted through a realistic avionics application, regarding the various PIs, in comparison to AFDX.

## 2. Background: Aircraft Data Communication Network

**Figure 1:**
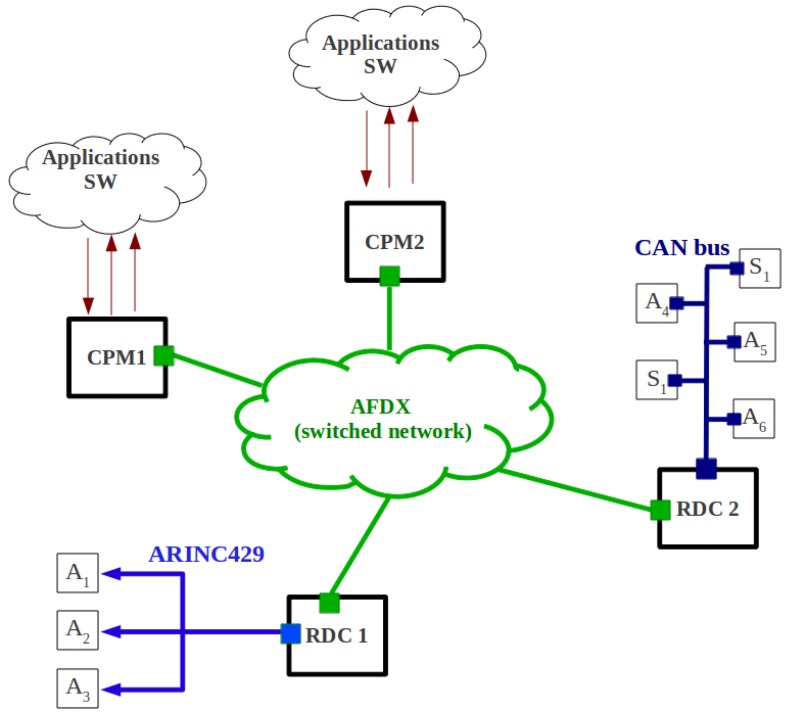
An Example of the Aircraft Data Communication Network [11].

As shown in Figure 1, the current ADCN consists of a backbone network, based on the AFDX, which interconnects the critical avionics subsystems. These subsystems are responsible for flight control, cockpit, engines and landing gears. However, some legacy systems for I/O and cabin management are still connected to low-rate buses. We describe herein these different networks and the main avionics requirements that have to be fulfilled by our proposed network.

### 2.1. Backbone Network: ARINC 664

The Avionics Full DupleX Switched Ethernet (AFDX), known also as ARINC 664 [2], is a 100 Mbits/s Ethernet-compliant technology, which has been certified to meet the main avionics requirements. The main concepts of such a technology are as follows: the Virtual Link (VL), the static data forwarding and the redundancy mechanisms.

#### 2.1.1. Virtual Link

The Virtual Link (VL) concept is a way to reserve a guaranteed bandwidth to each traffic flow. It uses a 16-bit value called a Virtual Link ID (VLID) to route the frames in the AFDX network. A VL is unidirectional and must always originate at one end-system and be destined to a predetermined set of subsystems (unicast or multicast). Each VL defines a bandwidth contract expressed in terms of (i) BAG (Bandwidth Allocation Gap), ranging in powers of 2 from 1 to 128 ms, which represents the minimal inter-arrival time between two consecutive frames; (ii) MFS (maximum frame size), ranging from 64 to 1518 bytes, which represents the size of the largest frame that can be sent during each BAG.

#### 2.1.2. Static Data Forwarding

**Figure 2:**
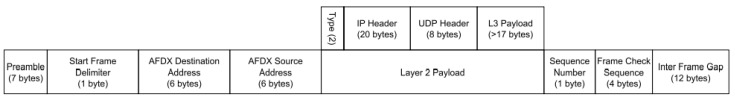
ARINC 664 frame structure.

AFDX uses the standard Ethernet frame where the destination MAC address field contains the VLID. In addition, there is a 1-byte sequence number to identify the redundant frames sent in the backup network. Figure 2 shows the structure of an AFDX frame. The end-to-end communication of a message using AFDX requires a static configuration of the source and destination end-systems and the AFDX switches.

#### 2.1.3. Redundancy Mechanisms

ARINC 664 specifies static redundancy mechanisms, based on two dedicated AFDX networks, i.e., a fully redundant network. Each subsystem is connected to an end-system to exchange messages on both AFDX networks, i.e., the end-system is the interface between an avionics subsystem and the AFDX interconnects. Each packet transmitted by an end-system is duplicated and sent on both networks. Therefore, if no transmission errors occur, each end-system will receive two copies of each packet. The first frame with a valid checksum to arrive at the end-system is consumed. Then, subsequent frames with a duplicate sequence number can be identified and discarded.

### 2.2. I/O Networks: ARINC 429 and CAN

#### 2.2.1. ARINC 429

Ref. [3] is one of the first standards specifically developed for avionics applications. It has been deployed in various avionics applications for decades and is still used in current aircraft as a low-rate I/O data bus.

ARINC 429 implements serial line communication. A line is a unidirectional and simplex connection, connecting one sending station LRU (Line Replaceable Unit) and multiple receivers (up to 19). A station may be attached to multiple buses and operates as either a sender or receiver, thus hierarchical layouts are possible.

ARINC 429 bus can operate at two transmission speeds: low or high speed. Low speed uses a variable clock rate with a throughput of 12–14 Kbits/s, while the high-speed mode requires a fixed clock rate and allows 100 kbps. The data unit transmitted in the medium is called a word and has a length of 32 bits. Various types of data are specified for the ARINC 429 communication, e.g., discrete and character data.

#### 2.2.2. Controller Area Network (CAN)

Ref. [5] is a broadcast digital data bus, designed in the 1980s by bosh for automotive applications, to replace the complex wiring harness with a two-wire bus. It was standardized by International Standard Organization (ISO) to provide 1 Mbits/s and 125 Kbits/s data rate for cable lengths up to 40 and 500 m, respectively.

CAN networks have been successfully used to replace point-to-point connections in many application domains, including avionics. This fact is due to its low cost, deterministic resolution of contentions, and error detection and retransmission mechanisms.

CAN supports two versions of protocols. The first uses the standard frame format with an 11-bit identifier, while the second uses an extended frame format with a 29-bit identifier. Since the bus is a shared medium, the connected controllers use the Carrier Sense Multiple Access with Collision Resolution (CSMA/CR) mechanism to avoid and solve collisions [12].

### 2.3. Requirements

The main avionics requirements concern both technical and cost aspects:

- High Rate: The number of embedded devices and functions is increasingly important, which increases the amount of exchanged data [1]. Hence, to cope with this growing expansion of the network, a high rate is required. Indeed, as with Moore’s law for processor power, the complexity of avionics systems doubles every 5 years, and to guarantee a long life for avionics systems (20 years on average), there is a need for a high rate to enable future development.
- Predictability: The network must behave in a predictable manner and guarantee maximum delays for any type of traffic. Thus, the system must be able to deliver accurate information within a bounded time. Moreover, avionics systems are hard real-time systems where critical messages need to be transmitted on time, even in the presence of non-critical messages. Then, a quality of service management must be provided.
- Modularity: This requirement is related to the flexibility and exchangeability of software and hardware components. An important step towards enhancing the avionics system modularity has been fulfilled with the adoption of the Integrated Modular Avionics (IMA) approach [13], i.e., common elementary components can be configured to fit different avionic applications. This feature aims to minimize the (re)configuration effort to facilitate system maintenance and its progress over the years. In the specific case of the AFDX, the implementation of an event-triggered paradigm minimizes the (re)configuration effort, thus favoring modularity.
- Reliability and Availability: The network must be fault tolerant and fulfill required reliability and availability levels to prevent failed nodes from affecting the normal operations. The reliability is *"the ability of a system to continuously deliver its intended services throughout a given interval of time"* [14]. In avionics, the backbone network must satisfy the Design Assurance Level-A (DAL-A) of the DO-254 [15] and DO-178 [16] documents. DAL-A is defined as “catastrophic”, where a failure may cause a crash, an error or the loss of critical function required to safely fly and land an aircraft and the failure rate must be less than $10^{-9}$/h. The availability metric concerns the recovery time which has been defined in [17] as *"the maximum time from failure to become fully operational again in case of a single permanent failure"*. For real-time applications, the network recovery time should be shorter than the grace time [17], which is about 20 ms for the most constrained real-time applications, i.e., time-critical automation or safety-critical avionics. For instance, to address these two metrics, redundancy mechanisms are implemented for the AFDX network to recover packet losses and faulty nodes during operation time and enable a null recovery time.
- Physical and electromagnetic resistance: Avionics devices are subject to severe physical constraints such as vibrations, the large range of temperature degrees and electromagnetic interferences. Therefore, the network must be very resistant physically and particularly at the level of connectors and cables.

Furthermore, the choice of an avionics network must be efficient to meet the design requirements for the least amount of money. Thus, the economic requirements are mainly as follows:

- Cost: Today, the communication network can reach 30% of the total cost of an aircraft, and this number will continue to grow. Thus, a good choice of avionics network is crucial to optimize the overall cost of the aircraft. The flexibility and configurability of components reduce development cycle duration, and ease incremental design and maintenance processes. Furthermore, the use of Commercial Off-The Shelf (COTS) technologies and the decrease of the quantity of wires infer a reduction in development and deployment costs, e.g., fuel consumption.
- IEEE 802.3 compatibility: To facilitate the adoption and the interoperability of the proposed network with the current one, i.e., AFDX.

## 3. Related Work: Improving ADCN Performance

Improving the performance of ADCN is still an ongoing task in academia and industry. There are several aspects related to such an objective, such as increasing the flexibility, resource efficiency, scalability, and reliability, while reducing complexity, heterogeneity and costs related to cabling, fuel consumption and integration. We identify herein two main classes in this specific domain.

The first class is based on wireless solutions [18,19] to reduce the weight and deployment costs. An interesting hybrid solution, denoted Wireless Safety-Critical Avionics Network (WSCAN), based on High Rate Ultra-Wideband (HR-UWB) [20] and switched Ethernet technologies, was proposed in [18] to replace the backup network of the AFDX backbone. The network consists of two clusters of subsystems interconnected through UWB technology. Then, the inter-cluster communication is enabled via gateways, connected to a Gigabit Ethernet switch. The choice of such a hybrid architecture is related to scalability issues. Moreover, the guarantees in terms of timeliness and reliability of such a solution have been proved under specific conditions of isolation from electromagnetic interferences. Despite the interesting advantages of using such a solution in terms of reducing weight and costs and increasing flexibility and efficiency, security is still a main challenge, due to its sensitivity to interference and jamming attacks [18].

The second class consists of either solutions working on the current ADCN architecture as in [21] or defining new architecture solutions as in [22]. In [21], a new CAN-AFDX Remote Data Concentrator (RDC) device was proposed to enhance the network bandwidth use, while meeting the timing constraints. Two major functions were adopted to reach this goal: (i) frame packing of the upstream flows coming from the CAN sensors, to reduce communication overheads, and consequently decreasing the AFDX bandwidth use; (ii) hierarchical Traffic Shaping (HTS) of downstream flows destined to actuators on CAN buses, to guarantee isolation between upstream and downstream flows on each I/O CAN bus, and consequently favoring the frame packing process. Although this solution improves the resource efficiency, it does not address the cost requirement, i.e., it does not reduce cabling-related costs.

In [22], the Time-Triggered Ethernet (TTE) was introduced as an embedded safety-critical Switched Ethernet network based on time-triggered communication [23]. It uses time scheduling, i.e., TDMA, with an off-line configuration to guarantee predictable and deterministic communication for the highest priority traffic, and it combines different types of data flows on the same network. Although TTE reduces heterogeneity and guarantees deterministic communication, it follows a completely different communication paradigm from the current AFDX network (which is based on event-triggered communication). The time-triggered characteristic will decrease the modularity level due to the need for synchronization, and increase the reconfiguration effort.

However, such an evolving issue (i.e., improving the performance of on-board communication systems) is not unique to avionics and is present also in other real-time applications, such as automation and automotive. For many of these applications, Ethernet technology is considered to be one of the most cost-effective solutions allowing scalable topologies and supporting high-speed communication. Furthermore, recent mechanisms for Ethernet are standardized or in the standardization process to improve the predictability and availability guarantees, such as Audio-Video Bridging (AVB) [24] and Time Sensitive Networking (TSN) [25], as well as to increase the resistance of the physical layer to harsh environment, such as 100BASE-T1 [26] and 1000BASE-T1 [27]. The latter was driven by the need to reduce the weight of wiring and increase the bandwidth. The 1000BASE-T1 is based on an unshielded single twisted pair at 1 Gbit/s and it was initially targeting automotive [28], then it also gained high interest in avionics for medium and low DALs, such as the cabin management [29,30].

Our proposal, AeroRing, is also a 1 Gbit/s Ethernet-compliant solution but it is targeting avionics for high DAL (DAL-A) and supporting ring topologies. The ring topology will enable further decrease in cabling complexity, in comparison to the switched one; thus, an inherent weight reduction and an increase of system efficiency, e.g., less fuel consumption. Moreover, it offers a high availability level due to the various redundancy solutions, which have been specified in the documents IEC62439-1/7 [17,31,32,33,34,35,36]. In fact, this topology provides an implicit redundant path by introducing only one additional connection between the two end nodes, compared to line or star topologies [37].

However, the main challenge for Ethernet-compliant solutions supporting a ring topology is reconciling the avionics requirements, while reducing the reconfiguration effort and deployment costs. To achieve this aim, we have specified AeroRing and assessed its performance indicators versus the avionics requirements as detailed in the following sections.

## 4. AeroRing Specifications

In this section, we first introduce the main features and supported topologies of AeroRing. Then, we describe the main mechanisms to guarantee QoS management and predictability. Finally, we present the specified ring redundancy protocol for AeroRing to guarantee availability, called QoS-aware Ring Redundancy Protocol (QoS-ARRP).

### 4.1. Main Features

AeroRing is a 1 Gbit/s multiple-ring Ethernet network that allows any “Ethernet-compliant” equipment to transmit its data in the network according to an event-triggered paradigm via a specific end-system, called T-AeroRing. The latter is the interface between an equipment and the AeroRing interconnects. Each transmitted packet will be forwarded from one T-AeroRing to another until reaching the destination.

**Figure 3:**
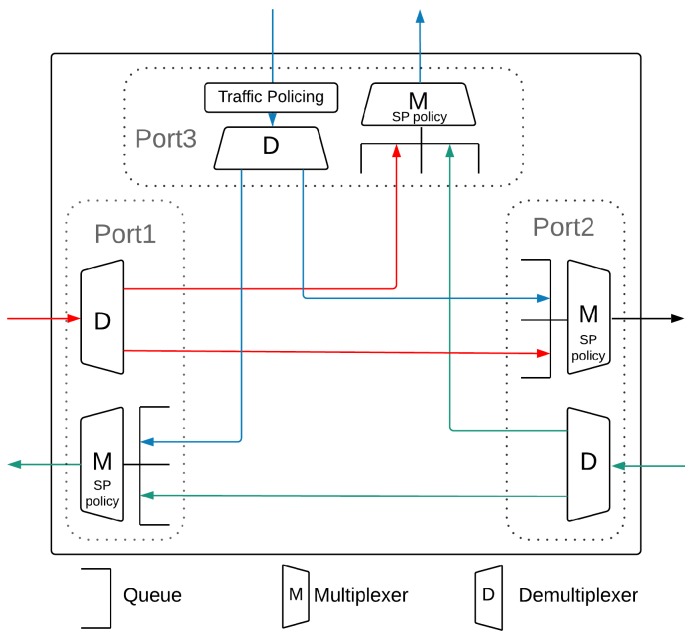
T-AeroRing internal architecture.

The T-AeroRing is a specific three ports Full DupleX Gigabit Ethernet switch, as illustrated in Figure 3. Ports 1 and 2 are used to transmit (resp. receive) data to (resp. from) the network, while Port 3 is used to transmit (resp. receive) data to (resp. from) the equipment. Each port consists of a *Demultiplexer* (D) to demultiplex received messages and forward them to the destination port, e.g., Port 3 if the destination is the connected equipment otherwise Port 1 or 2, and a *Multiplexer* (M) to multiplex the transmitted messages according to the implemented *service policy*. Moreover, Port 3 implements *traffic policing* mechanisms to avoid network saturation. The T-AeroRing has the following main characteristics:

- Cut-Through forwarding technique: The T-AeroRing starts forwarding the packet just after its identification, i.e., only the header of each packet is decoded to determine its destination port. This technique is implemented in the Demultiplexer and guarantees shorter transmission latency than the Store & Forward technique, which waits until the complete reception of the packet before forwarding it to the destination port.
- Class-based Strict Priority queuing: The T-AeroRing implements the class-based priority queuing defined in [38]. Packets are first classified according to their priorities and associated with a traffic class, to be then placed into different priority queues in each output port of the T-AeroRing (in the Multiplexer), i.e., one priority queue per class. A queue is selected for transmission only if all queues of higher priorities are empty. Then, within each of the priority queues, packets are scheduled according to First In First Out (FIFO) order. Four priorities are defined according to the IEEE 802.1p standard where the 802.1Q tag (3-bit field) is used to manipulate traffic classes: the control traffic class with the highest priority, the Hard Real-Time (HRT) class with the second highest priority, the Soft Real-Time (SRT) class with medium priority and finally the Non-Real-Time (NRT) class with the lowest priority.
- Traffic policing: To guarantee real-time performance, the T-AeroRing implements traffic policing mechanisms (in Port 3), based on the leaky bucket method as defined in [39] and particularly the greedy method [40,41], to control each traffic class compliance with its predefined contract to avoid the network saturation. These traffic contracts are characterized by an allocated bandwidth and a maximum burstiness depending on the network designer specifications. The leaky bucket method is based on a counter that is incremented whenever a packet is sent and decremented periodically according to the allocated bandwidth. If the counter exceeds a threshold (the maximum burstiness), then the packet is discarded. Each piece of equipment connected to a T-AeroRing should be aware of these traffic contracts and may apply traffic shaping to ensure the conformity of its generated traffic and avoid being discarded by the traffic policers.
- QoS-aware routing: Each T-AeroRing builds its routing table based on control messages exchanged between the interconnected T-AeroRings, during the initialization phase or when a topology modification occurs (i.e., failure or recovery), using the QoS-ARRP mechanism. Each T-AeroRing implements two routing modes to transmit its generated packets depending on their priorities: (i) sending on both ring ports (Ports 1 and 2 in Figure 3) for high priority traffic classes, i.e., control and HRT data, to allow a high availability level; (ii) sending on the port corresponding to the shortest path for medium and low priority traffic classes, i.e., SRT and NRT data, to offer short delay.
- QoS-Aware Ring Redundancy Protocol (QoS-ARRP): QoS-ARRP integrates dynamic mechanisms for fault detection and reconfiguration of routing tables, based on control messages. Moreover, QoS-ARRP implies low control overhead and enables the full use of ring topology, due to its filtering mechanisms that avoid infinite message looping.

### 4.2. Supported Topologies

**Figure 4:**
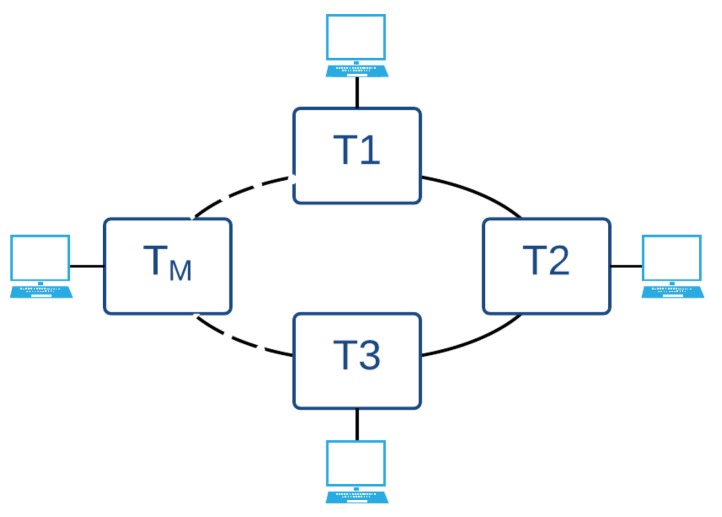
Mono-ring network architecture.

AeroRing supports several topologies. The first one is a ring daisy-chain implementation, called the simple mono-ring and illustrated in Figure 4. In this topology, T-AeroRings are connected in a daisy-chain mode using the ring ports, i.e., ports 1 and 2 in Figure 3, whereas port 3 is used to connect the communicating equipment.

**Figure 5:**
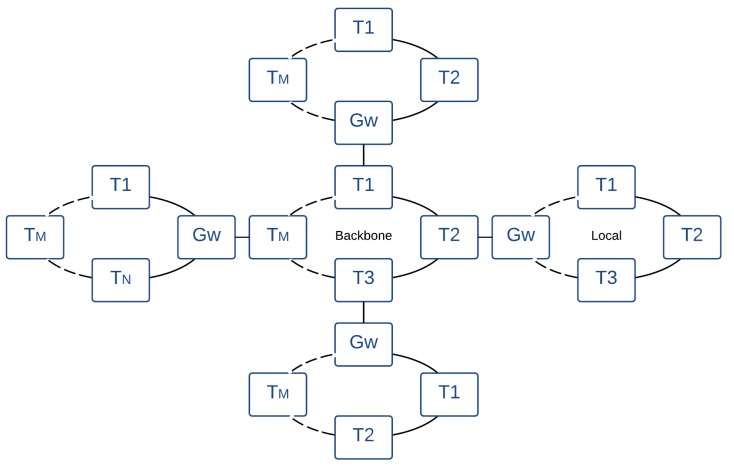
Multiple-ring network architecture.

The second AeroRing topology is the simple multiple-ring which connects different peripheral rings via a backbone ring, as shown in Figure 5. The key idea is to gather nodes in a peripheral ring according to their exchanged data to decrease the data path length. This fact will reduce the end-to-end delays and the fault detection and reconfiguration times, thus enhancing the availability level. Moreover, this topology may improve the throughput within each peripheral ring, since it isolates the intra-ring traffic from the inter-ring traffic.

The peripheral rings are connected to the backbone ring via gateways, which manage the inter-ring communications. The gateway is a specific T-AeroRing and its main function is guaranteeing the QoS-aware routing between the peripheral rings. This feature will be detailed in Section 4.3.

In addition to these two main topologies, AeroRing supports duplicated mono-ring and multiple-ring topologies. Each piece of equipment may be connected to redundant T-AeroRings and transmit its data on both AeroRing networks. The redundancy management for such topologies can be handled with classic static redundancy protocols, such as Parallel Redundancy Protocol (PRP) [36].

### 4.3. QoS Management and Data Processing

AeroRing guarantees QoS management through the implementation of “Class-based Strict Priority” queuing, which supports four traffic classes as follows:

1. Control traffic class: This traffic is generated at the T-AeroRing level to enable the QoS-ARRP mechanisms, e.g., fault detection and recovery as well as auto-configuration mechanisms, as detailed in Section 4.4. This traffic has the highest priority level (N0) to reduce the fault detection and recovery times, thus increasing the availability level of AeroRing.
2. HRT traffic class: This traffic has the second highest priority level (N1) and is generally generated by real-time applications, such as sensors, with hard temporal constraints. Each message must be received before its deadline, otherwise it is considered to be lost. This type of data flow is sent on both ring ports to ensure a high availability level and is identified by a 2-byte sequence number, essential to filter redundant messages within the destination T-AeroRing.
3. SRT traffic class: This traffic mainly sent by soft real-time applications, such as audio or video transfers, has the medium priority level (N2). This type of data flow is sent on the ring port corresponding to the shortest path to guarantee a high performance level, i.e., short transmission delay.
4. NRT traffic class: This traffic corresponds to non-real-time applications, such as file transfer, and has the lowest priority level (N3). This type of data flow is sent on the ring port corresponding to the shortest path to guarantee a high performance level.

**Figure 6:**
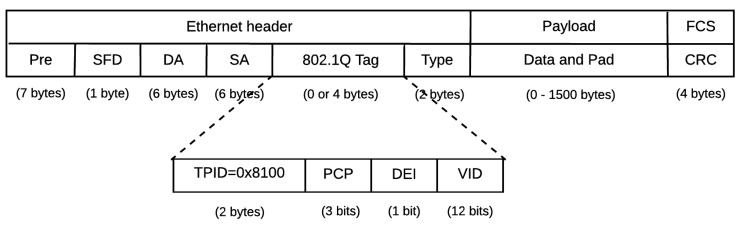
T-AeroRing frame structure.

The T-AeroRing priorities are handled according to the IEEE 802.1Q specification, as shown in Figure 6, where

- the 3-bit PCP field indicating the priority level of the message can encode up to eight levels. The mapping between the four priority levels of AeroRing and the standard eight priority levels is shown in Table 1;
- the VID field is used to identify the source peripheral ring of a message in the multiple-ring topology.

It is worth noting that the AeroRing is compatible with the IEEE 802.3 standard and each T-AeroRing can deliver any type of “802.3x-compliant” message sent by its equipment.

Hence, if the message does not include the 802.1Q tag, then it will be treated as a message of NRT data flow type (N3) and transmitted on the ring port corresponding to the shortest path.

Moreover, AeroRing enables two modes of broadcast:

1. A global broadcast to send data to all the network equipment. Such messages have a default VID value, i.e., 0x000. Furthermore, broadcast messages without the 802.1Q tag are sent following the global broadcast mode;
2. A local broadcast to send data only within a peripheral ring. Such messages have the VID of the corresponding peripheral ring.

The real-time behavior of AeroRing and the timeliness guarantee of the delivered data are favored due to the implemented features within the T-AeroRing. First, the “Cut-Through” forwarding technique allows a short transmission time along the network, which improves the Maximum end-to-end delivery time. Then, the traffic policing mechanism prevents network saturation caused by a deficient piece of equipment, which favors the communication determinism. Furthermore, the implemented QoS-aware routing algorithm supports the transmission of the SRT and NRT data flow on the shortest path, which decreases their transmission delays; whereas the HRT data flows are sent on both paths to increase the reliability level. Finally, the Class-based Strict Priority queuing ensures the temporal isolation between mixed criticality data with various temporal constraints and guarantees a bounded delay for the HRT traffic class.

### 4.4. QoS-Aware Ring Redundancy Protocol (QoS-ARRP)

**Figure 7:**
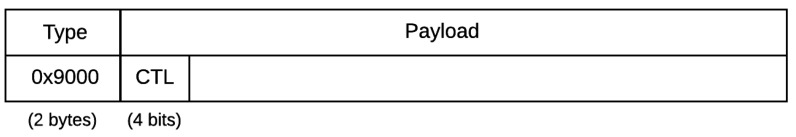
Sub-structure of a control message.

The QoS-ARRP protocol handles the frame redundancy and filtering and supports fault detection and reconfiguration mechanisms for the AeroRing network. These mechanisms are enabled through an exchange of control messages, which have the highest priority level (N0). Figure 7 shows the sub-structure of a control message, where the type value is ″0x9000″ and the CTL field identifies the type of the control message. In this section, we detail the main mechanisms of QoS-ARRP.

#### 4.4.1. Frame Redundancy

Based on the description of T-AeroRing ports in Figure 3, each message will be treated within an AeroRing node as follows:

1. Messages received from port 3, i.e., from the connected equipment, are transmitted to port 1 and/or 2 according to their priority level:
  - Messages with priority N1 (HRT) are sent through both ports;
  - Messages with priorities N2 (SRT) and N3 (NRT) are sent through the port corresponding to the shortest path to the destination if it belongs to the same peripheral ring; otherwise, to the gateway;
  - Broadcast messages with priorities N2 (SRT) and N3 (NRT) are transmitted through a predefined port or a port selected randomly.
2. Messages received from ports 1 or 2 are treated according to their priority level and destination address. If the destination address corresponds to the equipment connected to the T-AeroRing, then the message is sent to port 3; otherwise, the messages are forwarded to the opposite port. It is worth noting that each message with priority N1 (HRT) is sent to port 3 only if its replica has not been received yet.

On the other hand, the messages are treated within the gateway in a different way:

1. For messages received from a ring port, i.e., port 1 or 2, we distinguish three cases:
  - The 802.1Q-tagged (resp. non-802.1Q-tagged) messages are transmitted according to the VID (resp. MAC). If the VID corresponds to the peripheral ring VID (resp. the MAC is within the routing table of the gateway), then the messages are transmitted within the peripheral ring; otherwise, they are transmitted within the backbone ring;
  - The messages compliant with the global broadcast mode are transmitted within peripheral and backbone rings.
2. Messages received from the backbone (resp. peripheral) ring are transmitted according to the MAC (resp. VID). If the MAC (resp. VID) is within the routing table of the gateway or is a broadcast address, then the messages are transmitted within the peripheral (resp. backbone) ring, according to their priority levels similarly to a T-AeroRing; otherwise, messages are discarded according to the filtering rules detailed in Section 4.4.2. It is worth noting that for the non-802.1Q-tagged messages received from port 3, we have the same gateway behavior, except when the MAC address is not within the routing table of the gateway. In this particular case, they are transmitted within the backbone via a ring port selected randomly or a default one.

#### 4.4.2. Filtering Process

QoS-ARRP enables communication on both ring directions by implementing some filtering rules within the ring devices to avoid infinite message looping:

- For nominal transmission where both the message and its FCS field are correct, messages are filtered from the ring within the destination device or the source node, independently from their priority level. In addition, in the specific case of N1 messages, the destination node delivers only the first valid received replica. This fact is enabled due to the added sequence number of 2 bytes within the payload field, which allows the replicas to be identified and discarded. When the destination receives a correct N1 message, it stores the couple (src MAC, sequence number); and once the replica is received or after a timeout, the stored couple (src MAC, sequence number) is removed from the memory. It is worth noting that the timeout is a parameter fixed by the network designer, which must be greater than the maximum end-to-end delay.
- For non-nominal transmission where the FCS field is not correct, i.e., detected error, the filtering rules are similar to the previous case.
- For non-nominal transmission where the message is not correct and its FCS field is correct, i.e., non-detected error: if the error occurs on the header, then the frame can be eliminated from the network by any ring device based on its routing table, i.e., the source or the destination MAC address is not within the routing table.

On the other hand, messages are filtered within the gateways if the VID is not within the gateways routing tables. Furthermore, backbone gateways tag the messages with a TTL (Time To Live) field equal to the backbone size, which will be decremented by each gateway and removed when it reaches zero. This TTL field allows the filtering of global broadcast messages and non-802.1Q-tagged messages with an unknown (erroneous) source or destination MAC address.

#### 4.4.3. Fault Detection and Recovery

To reduce the control messages overhead on the network, QoS-ARRP uses a distributed local fault detection mechanism. A T-AeroRing deduces that its neighbor is operational if it receives any frame from it. Hence, any T-AeroRing must consider a connection as down with a neighbor, if it does not receive any message from its neighbor during a certain period called “detection period”. This detection period can be easily tuned by the network designer. Furthermore, the QoS-ARRP uses the link status information provided by the physical layer mechanisms of the Gigabit Ethernet, as specified in ISO/IEC 8802-3:20000 Clause 24.

Therefore, the QoS-ARRP failure detection mechanism is based on three main steps:

1. First, if a T-AeroRing has no data to transmit to its neighbor(s), then it announces periodically, each *announcing period*, its status to the neighbor(s) through sending control messages. These messages have the minimum Ethernet frame length of 64 bytes and are identified with a CTL field set to “0000” and a type value ″0x9000″, as illustrated in Figure 7.
2. Second, any T-AeroRing, which did not receive any data or control message from a ring port for a duration equal to the *detection period* (This period is higher than the *announcing period* and covers, in general, the reception of more than one control message), will detect a failure. Afterwards, this T-AeroRing informs the other T-AeroRings about the failure through a control message, which has the same structure as illustrated in Figure 7 and a CTL code “0010”.
3. Finally, any T-AeroRing receiving the control message with a CTL code “0010” from the T-AeroRing detecting the failure will update its routing table to bypass the failure during the data transmission. This step is based on the auto-configuration mechanism of the QoS-ARRP (described in Section 4.4.4).

Concerning the failure recovery process, when the T-AeroRing that has detected the failure starts receiving messages (data or control) from its neighbor again, then it deduces that the connection is operational again. Consequently, it sends a control message with a CTL code “0010” to enable the routing tables to update the other T-AeroRings, thus taking into account the failure recovery, based on the auto-configuration mechanism of the QoS-ARRP.

#### 4.4.4. Auto-Configuration Mechanism

To reduce the configuration effort for the network designer, the QoS-ARRP offers an auto-configuration service until all the network becomes operational, i.e., updated routing tables within all the T-AeroRings. Each ring performs its auto-configuration mechanism locally and independently from the other rings, i.e., backbone or peripheral.

This service is based on a simple address assignment method and a dynamic network topology discovery process. The address assignment of the connected T-AeroRings method consists in assigning the equipment address to its corresponding T-AeroRing, when it joins the network. This fact facilitates the communication between the connected equipment and avoids a heavy address translation step. However, each gateway keeps its predefined MAC address. Moreover, each peripheral ring admits a predefined VID.

**Figure 8:**
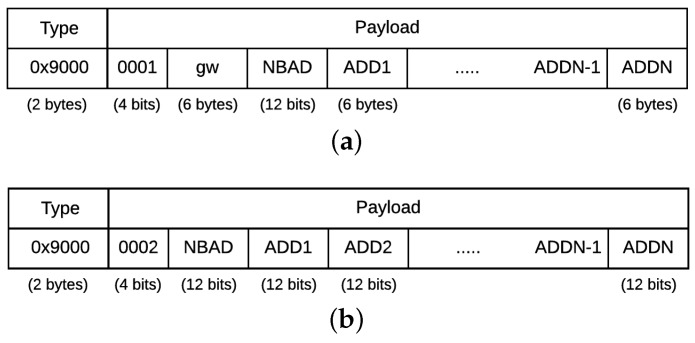
(**a**) Sub-structure of an auto-configuration control message within a peripheral ring; (**b**) Sub-structure of an auto-configuration control message within a backbone ring.

Messages are routed within the peripheral rings based on the MAC addresses and within the backbone ring based on the VID. Hence, peripheral T-AeroRings and gateway routing tables consist of the MAC addresses of the connected equipment, and the backbone gateways routing tables consist of the VIDs. These routing tables allow the selection of the port corresponding to the shortest path (ports 1 or 2) for a destination. They are built based on control messages exchanged between the nodes, i.e., T-AeroRings and gateways. The sub-structure of these control messages is illustrated in Figure 8.

Control messages used to build the routing tables in a peripheral ring consist of the following:

- CTL field set to “0001”;
- Gw field used by the gateway to specify its MAC address;
- NBAD (NumBer of ADdresses) field used as a counter of the addresses inserted in ADDx fields;
- ADDx (ADDresses) fields, used by the T-AeroRings to insert their addresses.

On the other hand, control messages used to build the routing tables in the backbone consist of the following:

- CTL field set to “0002”;
- NBAD field used as a counter of the VID inserted in ADDx fields;
- ADDx fields, used by the gateways to insert their VIDs.

The QoS-ARRP auto-configuration mechanism is based on the following main steps:

- First, any ring device, i.e., peripheral T-AeroRing or gateway, detecting a topology change event, i.e., equipment connection, device failure or recovery, sends periodically (The period can be tuned according to the application requirements by the network designer), on both ring ports, a control message with a NBAD field set to zero and empty ADDx fields, to update the routing tables of the other ring devices.
- Second, any ring device receiving such a control message will contribute in building/updating the routing tables through the following:
  1. updating the control message by incrementing the NBAD counter, inserting its MAC address at the end of the ADDx list to respect the physical order of the ring and computing the new FCS field;
  2. forwarding the updated control message to the next device;
  3. updating its routing table through inserting the MAC addresses of new devices and deleting the ones that no longer exist;
  4. Furthermore, the gateway inserts, in addition to its address in the ADDx, its address in the Gw field to enable its identification by other T-AeroRings.
- Third, when the device that has detected the topology change event receives such a control message on one of its ring ports, then it deduces that it has a neighbor on that port and it is no longer the last device of the segment. Consequently, it stops the periodic transmission of the control messages on the corresponding ring port, with a CTL “0001” (resp. “0002”) if it is within a peripheral (resp. a backbone) ring; and it stops completely transmitting control messages on both ring ports when detecting both neighbors.
- Finally, the transmission of such control messages stops completely when the ring is closed; thus, all the routing tables are up to date.

**Figure 9:**
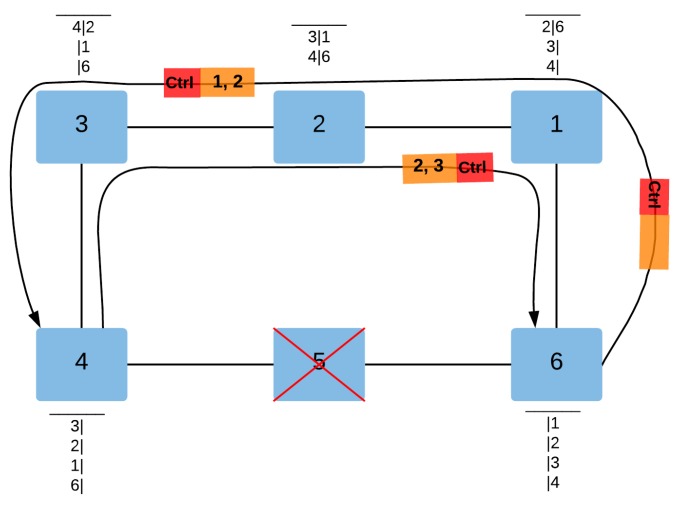
The QoS-ARRP Auto-configuration Mechanism after a failure detection.

**Figure 10:**
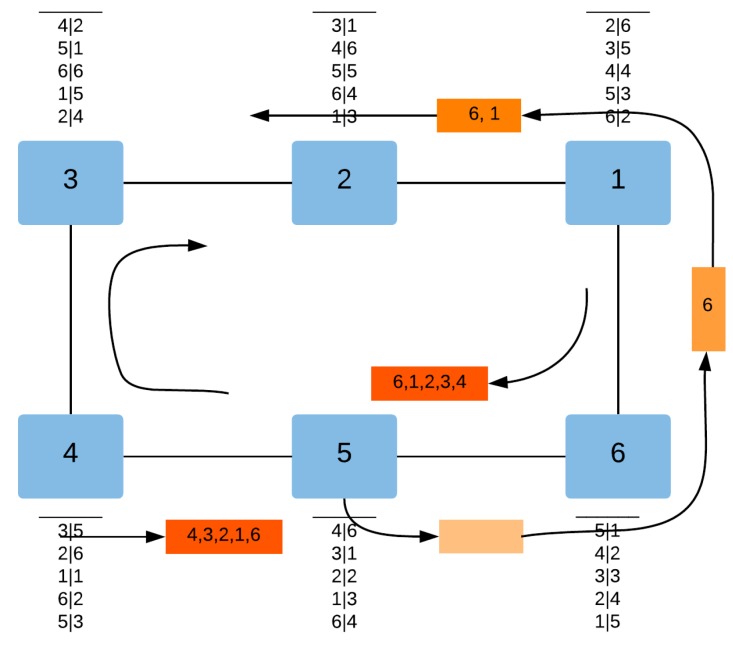
The QoS-ARRP Auto-configuration Mechanism after a failure recovery.

Figure 9 and Figure 10 illustrate the different steps of the QoS-ARRP auto-configuration mechanism for a failure detection event and a failure recovery event, respectively.

As shown in Figure 9, after the failure detection of the ring device 5 by the ring devices 4 and 6, the latter send control messages on both opposite ring ports, to inform the rest of the ring devices about the failure and to enable their routing tables to update. Afterwards, at the reception of the control message on a ring port, each ring device updates its routing table by erasing the MAC addresses located after the device detecting the failure, e.g., the device 3 erases all MAC addresses located after the device 4 (5,6,1,2) when receiving the control message on the corresponding ring port.

In Figure 10, after the failure recovery of the ring device 5, the latter sends control messages on both ring ports to enable the routing tables to update the other ring devices. Therefore, each ring device receiving the control message inserts its own MAC address, increments the NBAD field and updates the FCS. At the end, the device 5 receives the control messages on both its ring ports containing the list of MAC addresses to build its routing table.

### 4.5. Discussion

The main AeroRing features vs the avionics requirements described in Section 2.3 are summarized in Table 2:

- The choice of a Gigabit transmission capacity guarantees the *high rate* requirement.
- The “Cut-Through” forwarding technique, traffic policing mechanisms and QoS management (Class-based Strict Priority queuing and QoS-aware routing) reduce the end-to-end delays; thus enhance network *predictability*.
- The implementation of event-triggered paradigm and the distributed fault management protocol QoS-ARRP decrease the (re)configuration effort; thus increase the network *modularity*.
- The traffic policing prevents from the network saturation and the QoS-ARRP avoids any central point of failure; thus they improve the *availability* and *reliability* of the network.
- The choice of Ethernet technology guarantees the *IEEE 802.3-compliance* and the ring-based topology decreases the complexity of wires; thus the development and deployment *costs*.

Hence, as we can notice, AeroRing favors qualitatively the guarantee of the different avionics requirements. In the next sections, we will investigate these guarantees in a quantitative way.

## 5. AeroRing Performance Indicators

The second part of the standard IEC 61784 document [9] defines a set of Performance indicators (PIs) that allow the assessment of the ability of an Ethernet network to guarantee the needs of a real-time application. In this section, we detail the analytical formula of the main relevant PIs of AeroRing to measure its guarantees for avionic applications.

### 5.1. Delivery Time

The Maximum Delivery Time indicates “*the time needed to convey an Application Protocol Data Unit (APDU) containing data (message payload) that must be delivered in real-time from one node (source) to another node (destination)*” when considering the worst-case scenario. This PI is of utmost importance in avionics to conclude on the network *predictability*, since we need to guarantee that the maximum delivery time of any type of traffic is lower than its associated temporal deadline.

The maximum delivery time of AeroRing has been defined in [42] for mono-ring topology. We have applied the Network Calculus theory [40], which has actually been used for the certification of AFDX [43]. The main idea has consisted of solving the cyclic dependencies issue in AeroRing due to the ring topology and the specified event-triggered communication mechanism, i.e., there exist interfering flows with paths forming cycles. It is worth noting that the computation of this performance metric is not based on a closed-form formula as detailed in [42]. For the multiple-ring topology, there are actually two possible traffic flow patterns. The first pattern covers the intra-ring communication, i.e., exchanged flows between nodes within the same peripheral ring, and the maximum delivery time in this case is similar to the mono-ring case computed in [42]. The second pattern covers inter-ring communication, i.e., exchanged flows between nodes from different peripheral rings, and the end-to-end delivery time $D_{EED}$ in this case is the sum of the delivery times of crossed rings (the source peripheral ring, the backbone ring, and the destination peripheral ring). It is worth noting that the maximum delivery time of each crossed ring is similar to the mono-ring case computed in [42]. Therefore:

(1) $$D_{EED}=D_{src\_ring}+D_{backbone\_ring}+D_{dst\_ring}$$

where:

- $D_{src\_ring}$: The maximum delivery time within the source ring;
- $D_{backbone\_ring}$: The maximum delivery time within the backbone ring;
- $D_{dst\_ring}$: The maximum delivery time within the destination ring.

### 5.2. Maximum Number of End-Stations

The Maximum Number of End-stations in an AeroRing is computed as follows:

(2) $$Nbr_{end\_station}=\left\lceil \frac{Eth_{payload}-Ctl_{header}}{Add_{size}}\right\rceil$$

where:

- $Eth_{payload}$: Maximum Ethernet Payload size, which is equal to 1500 bytes for standard Ethernet frames and 9000 bytes for jumbo frames (enabled for Gigabit Ethernet);
- $Ctl_{header}$: The control message header, which is equal to 8 bytes (CTL, gw and NBAD fields) if the CTL is set to “001”, or 2 bytes (CTL and NBAD fields) if it is set to “002”.
- $Add_{size}$: The size of the conveyed addresses, which is equal to 6 bytes when the CTL is set to "001" (MAC address size) and “12 bits” (the VID size) if it is set to “002”.

Hence, a control message can contain up to 248 MAC addresses (resp. 998 VIDs) if we respect the maximum Ethernet payload size of 1500 bytes. Consequently, AeroRing with a mono-ring topology can support up to 248 nodes, and up to 998 peripheral rings with a multiple-ring topology.

Additionally, when using the jumbo frames (giant frames) that can go up to 9000 bytes, the mono-ring (resp. multiple-ring) topology can support up to 1498 nodes (resp. 4500 peripheral rings).

However, the guarantees on predictability and availability levels must be proved, when taking into account the characteristics of the transmitted flows.

### 5.3. Real-Time Throughput

The real-time throughput *"indicates the total amount of real-time APDU data (in bytes) on one link per second"*. This parameter allows the resource use efficiency of the network to be assessed, thus evaluating its maintainability during the long lifetime of an avionics system (about 20 to 30 years).

According to the real-time throughput definition, this indicator depends on the capacity of the link, the real-time flow rate transmitted by each node and the protocol overhead. Therefore, the real-time throughput of AeroRing is calculated by the following formula:

(3) $$Th_{RT}=\sum_{i=1}^{k}\left(APDU\_SIZE_{i}\times f_{i}\right)$$

where

- $APDU\_SIZE_{i}$: Payload data size of the flow *i*;
- $f_{i}$: transmission frequency of flow *i* equal to $\frac{\rho_{i}}{packet\_size_{i}}$ with $\rho_{i}$ the rate of the flow *i* and $packet\_size_{i}=APDU\_SIZE_{i}+overhead_{i}$;
- $overhead_{i}$: if the flow *i* is of HRT type, then the overhead is 44 bytes; otherwise, it is 42 bytes: 8 bytes of preamble, 12 bytes of IFG (Inter-Frame Gap), 12 bytes for the source and destination address, 4 bytes for the 802.1Q tag, 2 bytes for the type field, 4 bytes for the FCS field and 2 more bytes for the sequence number field for HRT frames;
- *k*: number of real-time flows through a link.

### 5.4. Non-Real-Time Bandwidth

The NRT Bandwidth *"indicates the percentage of bandwidth, which can be used for NRT communication on one link"*. This parameter can be considered as complementary to the real-time throughput indicator, to evaluate the effectiveness of the network in terms of resource use efficiency.

The NRT bandwidth is calculated based on the capacity of the link and the total real-time throughput (the percentage of the residual bandwidth). It is given by the following formula:

(4) $$B_{NRT}=\frac{R-\sum_{i=1}^{n}\rho_{i}}{R}=1-U_{RT}$$

where

- *R*: links capacity;
- $\rho_{i}$: rate of the flow *i*;
- *n*: number of real-time flows through a link, i.e., HRT and SRT flows;
- $U_{RT}$: real-time use rate.

### 5.5. Fault Detection Time

Fault Detection Time indicates “the maximum time needed to all nodes to be aware of failure”. This indicator is essential since it conditions the *availability* level of the network.

For AeroRing, a node detects a failure when it loses connection with its neighbor, or it receives a failure declaration by another node. The worst-case detection time in this case corresponds to the worst-case detection time of the node in the middle of the network according to the fault, i.e., the farthest node from failure in both directions. It is equal to the sum of the local detection time (detection of the failure by the neighbor) $T_{local\_detect}$, the transmission time of a control message to report the fault (64-bytes message) $T_{report}$, and the maximum delay while crossing the intermediate nodes $T_{delay}$. Hence, the fault detection time of AeroRing is as follows:

(5) $$T_{detection}=T_{local\_detect}+T_{report}+T_{delay}$$

To compute $T_{local\_detect}$, we consider that the local control messages are sent periodically, each $T_{local\_period}$, in the absence of traffic. Moreover, a node detects a failure after the loss of $N_{detect}$ local control messages. Hence, we have the following:

$$T_{local\_detect}=N_{detect}\times \left(T_{local\_period}+\epsilon \right)$$

$$T_{report}=\frac{L_{con}\times 8}{R}$$

where

- $L_{con}$: size of the control message (64 bytes for the minimum Ethernet frame size and 20 bytes for the preamble and IFG);
- *R*: links capacity.
- ϵ: technological latency.

To compute $T_{delay}$, we consider that the control message, which has the highest priority level (N0), can be delayed in the worst-case at each node by a maximum data packet length of low priority at each crossed T-AeroRing, due to the implemented non-preemptive class-based strict priority policy; thus,

$$T_{delay}=\left\lceil \frac{M-1}{2}\right\rceil \times \left(\frac{\max_{pp<7}L_{pp}\times 8}{R}+\epsilon \right)$$

where

- *M*: number of nodes;
- $L_{pp}$: maximum packet length with a priority pp strictly lower than the control message priority (7 as explained in Table 1);

### 5.6. Redundancy Recovery Time

Redundancy Recovery Time indicates *"the maximum time from failure to become fully operational again in case of a single permanent failure"*. This indicator is essential to evaluate the network availability, a key requirement in avionics.

For AeroRing, in the case of a failure, the HRT messages are always delivered to the destination since they are sent in both directions. Therefore, the recovery time for HRT type is null.

For other types of messages, $T_{recovery}$ is equal to the sum of (i) fault detection time $T_{detection}$, which is the maximum time needed for the neighbors of the faulty node or link to be aware of a failure; (ii) the delivery times of control messages for fault declaration $T_{decl}$ and routing tables update $T_{tab-up}$; (iii) the blocking delay due to low priority messages in each crossed T-AeroRing $T_{blocking}$ because of the non-preemptive strict priority policy. Therefore, the recovery time is as follows:

(6) $$T_{recovery}=T_{detection}+T_{decl}+T_{tab-up}+T_{blocking}$$

where:

- $T_{detection}$ is the local fault detection time and is computed in Equation (5);
- $T_{decl}$: is the transmission time of one control message of minimum size (64 bytes + 20 bytes) for fault detection;
- $T_{tab-up}=\frac{L_{adr-list}\times 8}{R}$ where $L_{add-list}$ is the length of the control message containing the list of MAC addresses (shown in Figure 8), used to update the routing table and is equal to 42+max(42,2+6×(M−3)) bytes, where 42 bytes is the overhead of the Ethernet header with the 802.1q tag including 12 bytes for the IFG, 2 bytes to identify the message type, and (M−3)×6 bytes is the size of an Ethernet MAC address multiplied by the maximum number of crossed nodes, i.e., all the nodes apart from the failed one and the two detecting the failure.
- $T_{blocking}=\left(M-3\right)\times \left(\frac{\max_{pp<7}L_{pp}\times 8}{R}+\epsilon \right)$.

## 6. Experimental Results

In this section, we evaluate the different Performance Indicators of AeroRing under different scenarios. First, we conduct a sensitivity analysis of those Performance Indicators, when considering a mono-ring AeroRing configuration while varying the network size. Then, we consider a representative avionics backbone network of an A380 based on the AFDX standard [2], where we evaluate the predictability and availability of AeroRing, in comparison to the AFDX.

### 6.1. Sensitivity Analysis

We consider the case study with the following assumptions:

- The network topology is a simple ring;
- The link speed is 1 Gbit/s;
- The network size varies from 5 to 100 nodes with a step of 10;
- All devices are similar and send the same traffic in broadcast mode;
- Each device generates three flows, one per each type of traffic as described in Table 3: the I/O data initially transmitted on the CAN and ARINC 429, the legacy AFDX flows and file transfer data for monitoring.

**Figure 11:**
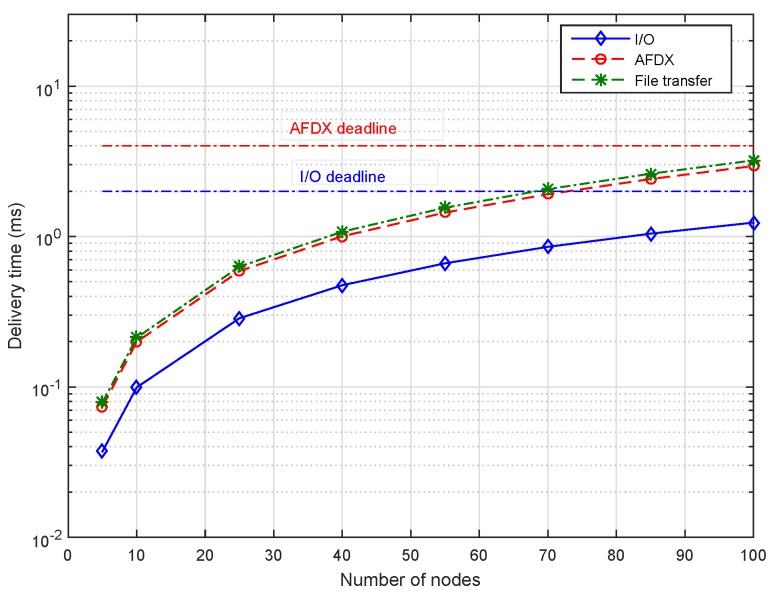
Maximum Delivery Times of AeroRing vs the network size.

Figure 11 illustrates the maximum delivery time of AeroRing for the different traffic classes when increasing the network size. We observe that AeroRing is highly scalable since it allows the connection of up to 100 end-systems, while respecting the most constrained deadline, i.e., I/O deadline of 2 ms. Moreover, the delivery times are about 1.24 ms, 2.9 ms and 3.2 ms for the I/O, the AFDX legacy and File Transfer traffics, respectively. These results show the high predictability level of AeroRing, mainly due to its QoS management mechanisms.

**Figure 12:**
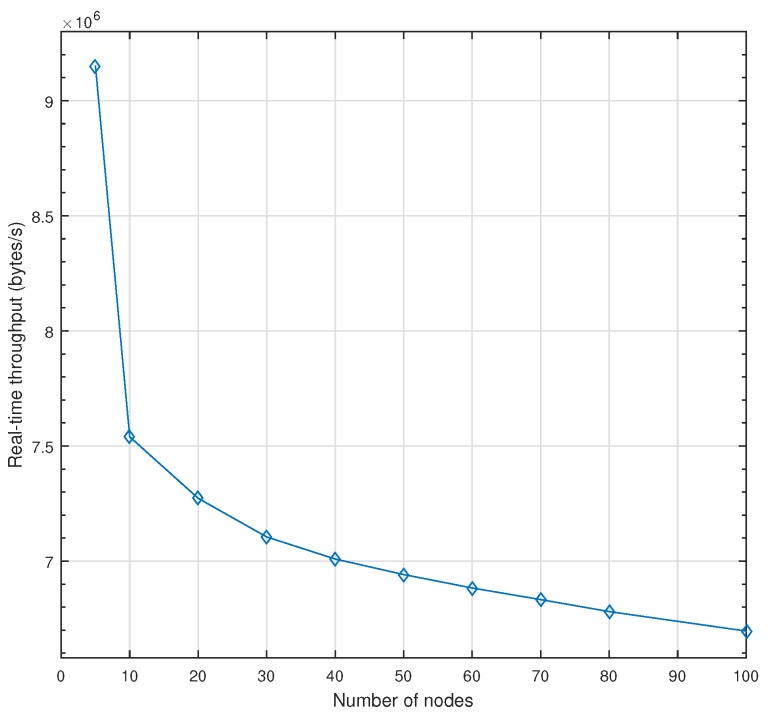
Real-time Throughput of AeroRing vs the network size.

Figure 12 illustrates the real-time throughput of AeroRing when increasing the network size. This metric has been computed when considering only I/O data (HRT class) as real-time traffic through a link in Equation (3). The idea consists in increasing the frequency of I/O data until violating its deadline constraint (2 ms) while keeping the payload size equal to 8 bytes for each considered network size. The main observation to note from this analysis is that the real-time throughput of AeroRing decreases in a noticeable way with the network size but it still decreases logarithmically. For instance, when the network increases from 10 to 100 nodes, i.e., ×10, the real-time throughput decreases only from almost 60 Mbit/s to 54 Mbit/s, i.e., ×0.1. These results infer that increasing AeroRing scalability has a limited impact on its real-time throughput. This fact shows the high resource efficiency of AeroRing.

**Figure 13:**
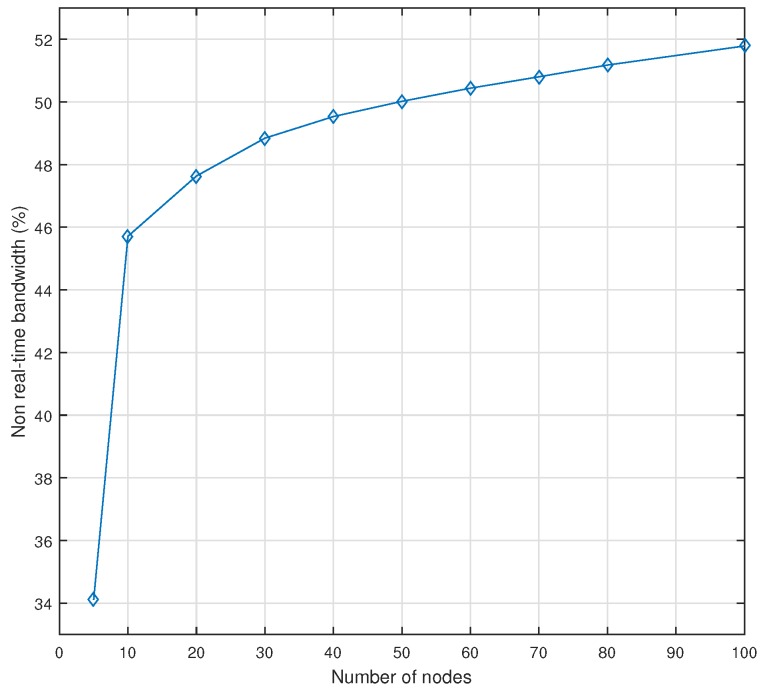
Non-real-time Bandwidth of AeroRing vs the network size.

On the other hand, Figure 13 illustrates the NRT bandwidth of AeroRing when increasing the network size. As we can notice, this metric and the real-time throughput are complementary, and both have been computed under the same scenario. The results confirm the stability of AeroRing in terms of resource efficiency, since we can use 46% of the capacity to send NRT traffic for a network of 10 nodes and achieve 52% for a network of 100 nodes.

It is worth noting that these values are computed when considering the residual capacity of the network after serving the maximum rate of real-time traffic, as explained in Equation (4). The maximum rate is nine times higher than the real-time throughput shown in Figure 12, since we are accounting for the protocol overhead, i.e., the frame length of an I/O data is 72 bytes for a payload of 8 bytes.

**Figure 14:**
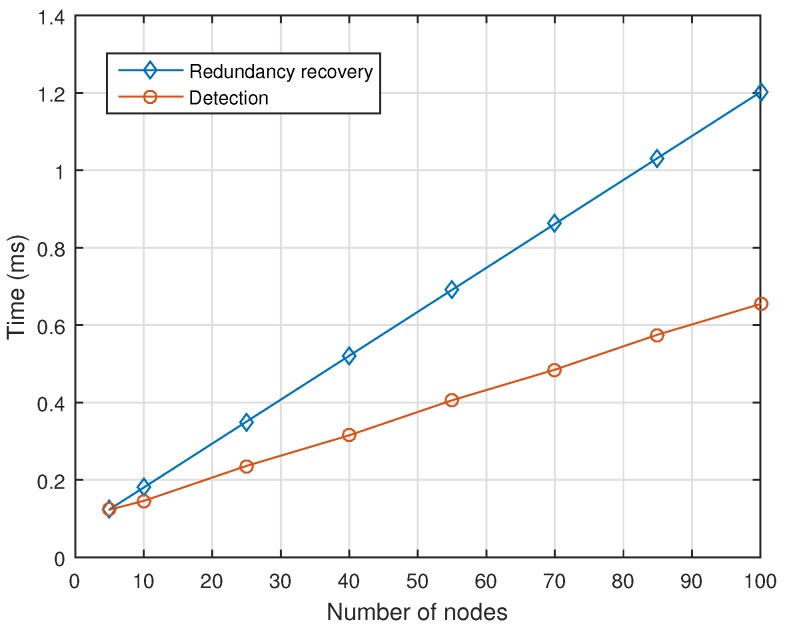
Redundancy Recovery and Detection Times of AeroRing vs the network size.

Finally, to assess the availability level of AeroRing, the maximum redundancy recovery and fault detection times of AeroRing are computed for different network sizes and the results are illustrated in Figure 14. It is worth noting that these results concern only the SRT and NRT types, since the HRT data are duplicated, and their redundancy recovery time is null. As we can notice, the maximum recovery time for a network size of 100 nodes is 1.2 ms, which is less than the most constrained deadline, i.e., I/O deadline of 2 ms, and less than the grace time [17] for the most constrained real-time applications, i.e., 20 ms. This highlights the high availability level of AeroRing. This fact is mainly due to the efficient fault detection and reconfiguration mechanisms of the QoS-ARRP, which allow fast fault detection and reconfiguration, i.e., updating routing tables using a single control message.

The conducted sensitivity analysis of AeroRing Performance Indicators regarding the network size has shown low delivery and recovery times as well as high real-time throughput and NRT bandwidth for large-scale network. This fact highlights the high predictability, availability, and resource-efficiency levels of AeroRing, with reference to avionics requirements.

### 6.2. Avionics Case Study

**Figure 15:**
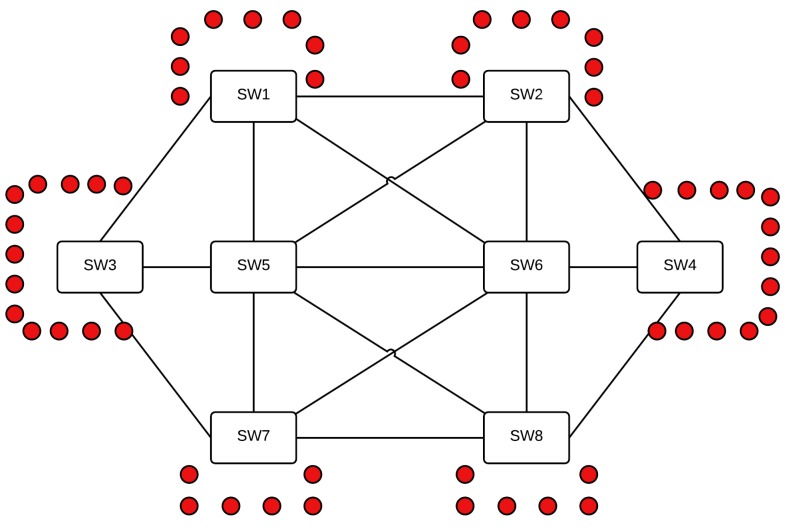
A representative A380 AFDX network.

The considered case study is a representative avionics backbone network of an A380, provided by Airbus during a collaborative project in 2001 aiming to certify the AFDX network [43]. As shown in Figure 15, it consists of eight AFDX switches connecting 54 end-systems, with six switches connecting between six and 13 end-systems each, and two additional switches connecting the other switches to reduce the number of hops of exchanged data between any two end-systems. Table 4 summarizes the described configuration in terms of number of end-systems and Virtual Links (VLs) for each switch.

The traffic characteristics of this AFDX configuration are shown in Table 5. There are three different types of traffic: the first one has a BAG value of 4 ms and MFS value of 480 bytes and is of HRT class; the second has a BAG value of 8 ms and MFS value of 16 bytes and is of SRT class; and the third has a BAG value of 32 ms and MFS value of 480 bytes and is also of SRT class. Moreover, each end-system generates 8 VLs.

To investigate the AeroRing performance, we replace the current AFDX backbone network described in Figure 15 and Table 4 by an AeroRing network. Then, we compare the computed delivery times, with reference to the AFDX network. It is worth noting that the current AFDX is a 100 Mbit/s network. Hence, to conduct fair comparative analysis, we enlarge its speed to 1 Gbit/s as AeroRing.

**Figure 16:**
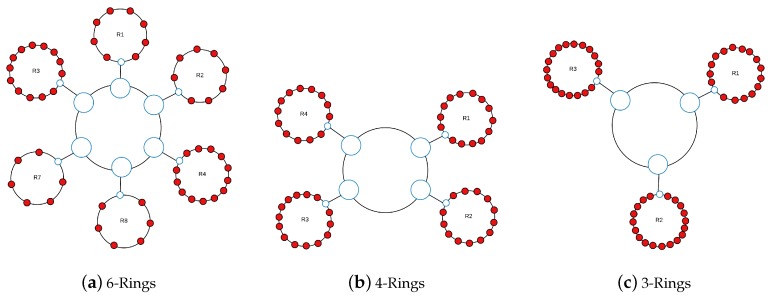
Considered Multiple-Ring Topologies for the Avionics Case Study.

The considered AeroRing topologies are described in Table 6 and illustrated in Figure 16:

- The 6-ring topology, described in Figure 16a and Table 6, where we replace each AFDX switch by a peripheral ring.
- The 4-ring topology as described in Figure 16b and Table 6, where switches SW3 and SW4 are each replaced by a peripheral ring, whereas switches SW1 and SW7 (resp. SW2 and SW8) are grouped within the same peripheral ring.
- The 3-ring topology as described in Figure 16c and Table 6, where each couple of switches among (SW1, SW2), (SW3, SW7) and (SW4, SW8) is replaced by one peripheral ring.
- The mono-ring topology, where all the end-systems of the AFDX switches are gathered in the same ring.

**Figure 17:**
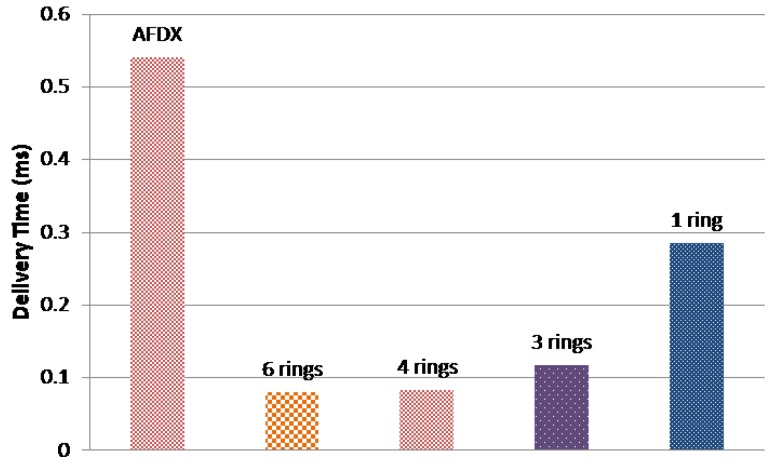
Maximum delivery times for traffic class HRT.

**Figure 18:**
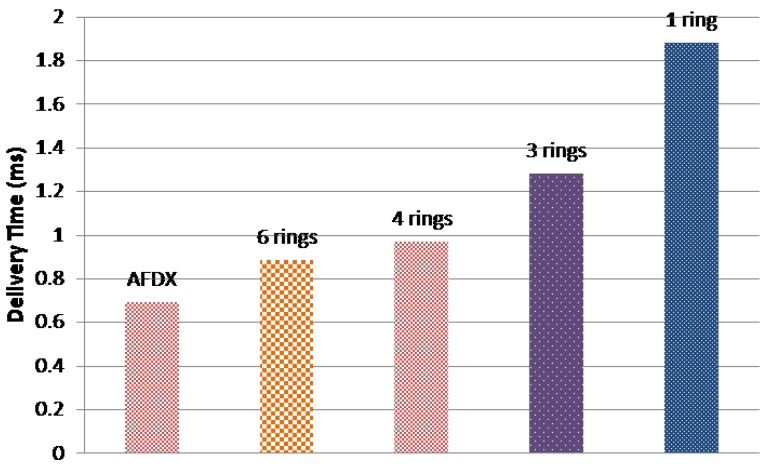
Maximum delivery times for traffic class SRT.

Figure 17 and Figure 18 show the maximum delivery times of the current AFDX network and the different AeroRing configurations for the different traffic classes. As we can notice, all solutions respect the temporal constraints of the different traffic classes. In addition, AeroRing offers lower delay for HRT traffic than AFDX. For instance, 6-ring AeroRing offers a delay bound for HRT 6.75 times less than the AFDX. These results show the high timing performance of AeroRing, with reference to AFDX.

**Figure 19:**
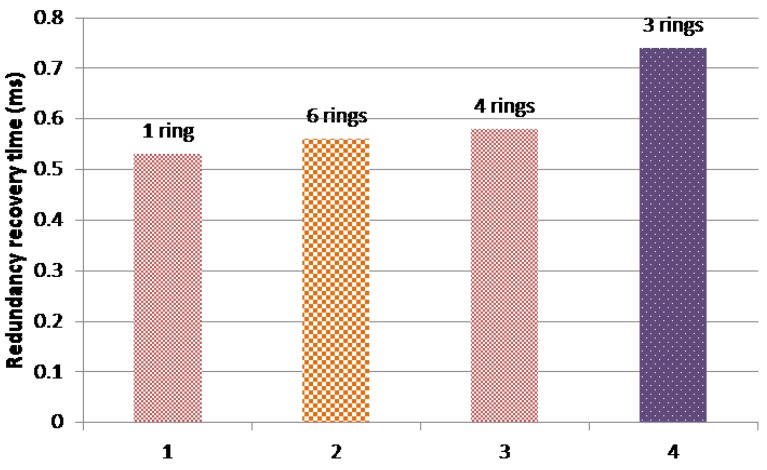
Maximum recovery time of AeroRing under various topologies.

In addition, Figure 19 shows a comparison of the maximum recovery time of the different AeroRing configurations. The AFDX and AeroRing offer null redundancy recovery times for the HRT class, since this data type is duplicated in both networks. The reported results of AeroRing concern only the SRT class. As we can see, the different AeroRing configurations offer a low recovery time, e.g., less than 1 ms which is well below the grace time of 20 ms. This result shows the high availability of AeroRing, which is mainly due to the QoS-ARRP protocol.

Moreover, we notice that the delivery and recovery times of AeroRing increase when reducing the number of peripheral rings, i.e., increasing the peripheral ring size. This fact is due to the increasing number of crossed nodes when the peripheral ring size increases, which increases the interferences. Hence, the 6-ring AeroRing configuration is the most efficient one to replace the current AFDX network.

## 7. Conclusions

To meet the emerging needs of the current ADCN and overcome its identified limitations, we have presented a new avionics communication network, AeroRing, based on the Gigabit Ethernet technology and a ring-based topology, which has the following advantages:

- Guaranteeing an easy deployment process and a cost-effective integration due to its IEEE 802.3 Compatibility and enabling various ring-based topologies, i.e., simple or duplicated mono-ring and multiple-ring topologies, based on auto-configuration mechanisms.
- Providing a high modularity level and reducing the (re)configuration effort, through implementing an event-triggered communication paradigm.
- Favoring predictability using Class-based Strict Priority queuing, QoS-aware routing algorithm and traffic policing mechanisms, to handle heterogeneous data constraints.
- Offering a high availability level due to the QoS-ARRP Protocol, which reduces fault detection overheads and increases the network use rate.

Afterwards, the AeroRing performance, i.e., timing and availability levels, has been assessed through a realistic avionics use case. The results have shown that the 6-ring AeroRing configuration is an interesting solution to replace the current AFDX network.

In the future, we plan to extend the application domains of AeroRing, specifically for a smart factory. This extension will entail new challenges in terms of scalability and adaptability features.

## Figures and Tables

**Table 1:** AeroRing priority levels.

Field	Priority level	Data type	AeroRing Priority
000	0	Best effort	N3
001	1	Background	N3
010	2	Excellent effort	N3
011	3	Critical App	N2
100	4	Video	N2
101	5	Audio	N1
110	6	Internetwork	N1
111	7	Network control	N0

**Table 2:** AeroRing features vs. avionics requirements.

Requirements	High Rate	Predictability	Modularity	Availability	Reliability	Cost	IEEE802.3 Compatibility
1 Gbit/s Ethernet	√					√	√
Ring-based topology						√	
Event-triggered paradigm			√				
Cut-through		√					
Strict Priority		√					
Traffic policing		√			√		
QoS-Aware routing		√					
QoS-ARRP			√	√	√		

**Table 3:** Traffic Characteristics.

	Priority	MFS (byte)	BAG (ms)	Deadline (ms)
I/O data	HRT	8	2	2
Legacy AFDX	SRT	1300	10	4
File Transfer	NRT	160	20	infinity

**Table 4:** Description of the AFDX configuration.

Switch ID	# End-Systems	# VLs
SW1	8	64
SW2	8	64
SW3	13	104
SW4	13	104
SW5	0	0
SW6	0	0
SW7	6	48
SW8	6	48

**Table 5:** Traffic Classes.

TC	Period (ms)	Payload Size (byte)	Rate (bit/s)	# VLs/End-System
HRT	4	480	$1024\times 10^{3}$	1
SRT	8	16	$72\times 10^{3}$	1
SRT	32	480	$128\times 10^{3}$	6

**Table 6:** Multiple-ring configurations.

Peripheral Ring Id	6 Rings	4 Rings	3 Rings	1 Ring
R1	SW1	SW1+SW7	SW1+SW2	SW1+SW2+SW3+SW4+SW7+SW8
R2	SW2	SW3	SW4+SW8	-
R3	SW3	SW2+SW8	SW3+SW7	-
R4	SW4	SW4	-	-
R5	SW7	-	-	-
R6	SW8	-	-	-

## Abbreviations

The following abbreviations are used in this manuscript:

A300	Airbus A300
IMA	Integrated Modular Architecture
AFDX	Avionics Full DupleX Switched Ethernet
HRT	Hard Real-Time
SRT	Soft Real-Time
NRT	Non-Real-Time
QoS	Quality of Service
S-ARRP	Quality of Service-Aware Ring Redundancy Protocol
ADCN	Aircraft Data Communication Network
PI	Performance Indicator
VL	Virtual Link
VLID	Virtual Link ID
ES	End-System
BAG	Bandwidth Allocation Gap
MFS	Maximum Frame Size
LRU	Line Replacement Unit
ISO	International Standard Organization
CSMA/CD	Carrier Sense Multiple Access with Collusion Detection
CSMA/CR	Carrier Sense Multiple Access with Collusion Resolution
DAL	Design Assurance Level
COTS	Commercial Off-The Shelf
WSCAN	Wireless Safety-Critical Avionics Network
HR-UWB	High Rate Ultra-Wideband
HTS	Hierarchical Traffic Shaping
TTE	Time-Triggered Ethernet
PRP	Parallel Redundancy Protocol
TTL	Time To Live
GW	GateWay
NBAD	NumBer of ADdresses
